# Pictorial Campaigns on Intimate Partner Violence Focusing on Victimized Men: A Systematic Content Analysis

**DOI:** 10.3389/fpsyg.2020.01450

**Published:** 2020-07-09

**Authors:** Eduardo Reis, Patrícia Arriaga, Carla Moleiro, Xavier Hospital

**Affiliations:** ^1^Social and Organizational Psychology Department, CIS-IUL/ISCTE-Instituto Universitário de Lisboa, Lisbon, Portugal; ^2^Inclusive Policy Lab, UNESCO, Dakar, Senegal

**Keywords:** intimate partner violence, victimized men, pictorial campaigns, prevention, help-seeking

## Abstract

Men who are victimized in their intimate different-sex (DS) and same-sex (SS) relationships often report not having information to help them escape their abusive situations. To overcome this lack of information, public awareness campaigns have been created. But thus far, there is no clear understanding of how these campaigns reflect theoretical principles central to improve message effectiveness and avoid undesired negative effects. This study aims to review the content of intimate partner violence (IPV) pictorial campaigns focusing on victimized men in DS and SS relationships. Specifically, it aims to understand the campaigns’ global characteristics and if their content represents constructs from different theoretical models. Online search engines were used to extract pictorial campaigns in English, Spanish, and Portuguese, released up until 2019. They must have had to be promoted by a formal organization, and were coded according to a theoretically grounded taxonomy, using thematic analysis. Our results indicate that out of the 57 campaigns collected, most were aimed at men without specifying the relationship they were in (i.e., DS or SS) (*n* = 22, 39%) and intended to change attitudes, beliefs, and behaviors about IPV (in line with the Theory of Planned Behavior) (*n* = 47, 82%). Additionally, four campaigns adequately integrated fear appeal constructs of the Extended Parallel Processing Model (*n* = 4, 7%), while 41 campaigns highlighted dissonant states in line with the Elaboration Likelihood Model (*n* = 41, 72%). Following the Transtheoretical Model, most campaigns targeted victims in the Maintenance stage (*n* = 52, 92%). The campaigns under analysis may prove useful for some victimized men, mostly presenting messages designed to elicit a beneficial attitude and behavior change. Our analysis highlights different limitations as well, such as the lack of information on susceptibility to IPV (*n* = 13, 23%) and the effectiveness of the recommended responses that the campaigns provide (*n* = 20, 38%), which may interfere with adequate fear appeal processing. Additionally, presenting more diverse victims may be beneficial, along with social norms change information regarding gender roles, violence, and help-seeking. This may guide the development of improved and tailored campaigns to better facilitate help-seeking in victimized men that mostly avoid undesired negative effects on the viewer.

## Introduction

The literature on intimate partner violence (IPV) and domestic violence has traditionally focused on female victims and male perpetrators. However, an ever-increasing number of studies have focused on men’s victimization, considering violence that occurs in different-sex (DS) (Cook, 2009) and same-sex (SS) relationships (Edwards et al., 2015b). Evidence suggests that victimized men in DS and SS relationships suffer significantly in their abusive relationships (Nowinski and Bowen, 2012) and encounter specific difficulties when recognizing the violence and seeking help (Hines and Douglas, 2011).

Victimized men face many internal barriers (e.g., shame, guilt), and they tend to not recognize themselves as victims due to masculinity norms, by which they are expected to be strong and emotionally controlled (McClennen et al., 2002). Additionally, gay, bisexual, transgender, and intersex (GBTI) men may have to disclose their sexual orientation and/or gender identity when seeking help (Edwards et al., 2015b). Some external barriers have also been reported, such as the lack of knowledge about the available support services (Machado et al., 2016). To overcome these barriers and increase help-seeking, IPV awareness campaigns have been created. The available research on this field indicates that female victims have perceived IPV awareness campaigns targeting them as emotionally harmful and inaccurate, given that generally, they depicted explicit physical violence and lacked empowering information for the victims (West, 2013). These perceived negative effects could potentially be overcome by following the literature on effective campaign design. However, to the best of our knowledge, there is no research addressing the impressions of victimized men in DS/SS relationships on campaigns focusing on victimized men, highlighting the need to conduct research focusing the specific realities of these relationships and on how to develop campaigns to avoid these unintended effects.

### Principles of Campaign Design

Over the last decades, research on the use of fear appeals for health promotion points to their effectiveness in positively influencing attitudes, intentions, and behaviors (Tannenbaum et al., 2015). Fear appeals have been defined as messages that elicit fear by highlighting the negative consequences of not doing a certain behavior (Witte et al., 2001). Adequately constructing and presenting these fear appeals are key to potentiate their effectiveness (Witte et al., 2001; Peters et al., 2013), as negative unintended effects may occur and audiences can react unpredictably to fear appeal messages (Witte et al., 2001; Cho and Salmon, 2007). One of these unintended effects is the boomerang effect, in which a message can potentially reinforce undesired attitudes and behaviors due to not being adequately processed (Witte et al., 2001). In order to avoid this, campaigns should be tailored to the social and psychological profiles of the target audience (Maibach and Cotton, 1995) and be based on previously tested theoretical approaches and constructs (Noar, 2006). This is consonant with a “contingency effects approach,” which states that message effects can depend on numerous factors that should be taken into account when developing campaigns and evaluating their effects (Riffe et al., 2005). Thus, including different theoretical approaches may be beneficial, as the consideration of the different processes inherent to message design, its cognitive processing, and behavior change adequately frames the viewer’s experience and may also lead to an adequate assessment of the campaigns’ expected effects (Witte et al., 2001). This could lead to more effective IPV campaigns that could facilitate beneficial attitude and behavior change, potentially leading to more recognition of violence and help-seeking attitudes and behaviors.

### Theoretical Frameworks

In the present study, we analyzed the content of campaigns focusing on victimized men in DS/SS relationships by considering four theories that have often been used to study the effectiveness of campaigns and the use of fear appeals. More specifically, we considered the Extended Parallel Processing Model (EPPM) (Witte et al., 2001), which is specific to fear appeals and campaigns, and the Theory of Planned Behavior (Ajzen, 1991), the Elaboration Likelihood Model (Petty and Cacioppo, 1986), and the Transtheoretical Model (Prochaska and Velicer, 1997) that are more broad in their applications but can be included in campaign design and the assessment of its effects.

The EPPM (Witte et al., 2001) suggests that, when faced with health threat messages, viewers firstly appraise information regarding threat severity and susceptibility, and then self-efficacy and efficacy of the recommended response. Threat severity corresponds to the significance or seriousness expected from a threat, while threat susceptibility is the likelihood that a specific target will experience the threat. Self-efficacy represents the degree to which the target perceives that he/she is able to perform the recommended response to avert the threat. And finally, response efficacy corresponds to the degree to which the recommended response effectively averts the threat. According to the model, in order to adequately process a fear appeal, it should be able to elicit a sufficient level of threat susceptibility and severity, but an even higher perceived self-efficacy and efficacy of the recommended response (Witte et al., 2001). Otherwise, the viewer may not pay attention or may reject the message. Applications of the EPPM to the IPV domain have been scarce, but some evidence suggests the utility of this model in understanding how important its constructs are for the development of IPV campaigns (Keller and Honea, 2016).

The Theory of Planned Behavior (Ajzen, 1991) provides important insights into behavior intention development. According to this model, the emergence of an intention is the result of the combination of attitudes toward the behavior, subjective norms about the behavior, and perceived behavioral control. When applied to health promotion, this model suggests that to generate actual behavioral change, practitioners should target all these components, given that intention strength will be greater when all are adequately framed (Ajzen, 1991). The Theory of Planned Behavior has been shown to be a suitable framework to understand different behavioral intentions in the context of IPV and domestic violence (Betts et al., 2011), both on intentions to leave the abusive relationship (Byrne and Arias, 2004; Edwards et al., 2015a) and intentions to perpetrate violence (Tolman et al., 1996; Kernsmith, 2005).

The Elaboration Likelihood Model (Petty and Cacioppo, 1986) adds that persuasion and possible attitude change can occur through two main routes. If the target is motivated and capable to process the message, the person will process it through the central route. Persuasion can still occur through a peripheral route in case these conditions are not met. In these cases, the target will focus on more contextual cues to process the message and will possibly be persuaded. It is important to note that persuasion does not have to occur solely through one of these routes, and that the role of peripheral cues (e.g., attractiveness, credibility, similarity of the source of the message to its viewer) is crucial to capture viewers’ attention (Petty and Cacioppo, 1986). Additionally, dissonance states may play a role in cognitive elaboration when processing a message, given that the viewer often aims to understand and dissolve the dissonance (Prunty and Apple, 2013). This model has been previously used in the development of an IPV campaign for domestic violence prevention, demonstrating preliminary positive results (Keller and Otjen, 2007), and has been noted to have an adequate framework for content analysis in campaigns in other fields (Igartua et al., 2003).

Finally, the Transtheoretical Model aims to explain the readiness for behavior change (Prochaska and Velicer, 1997). Specifically, it states that behavior change occurs through the following series of stages: Pre-Contemplation, Contemplation, Preparation, Action, Maintenance. In the Pre-Contemplation stage, individuals do not have intentions to change their behavior. The reason for this lack of intention to change may be due to not being aware of the behavioral options available or denial of the situation they are in. In the Contemplation stage, individuals begin to contemplate the need for change and start realizing the risks associated with a given behavior. In the Preparation stage, individuals commit to changing and prepare for an eventual behavioral change. In the Action stage, individuals perform the new behavior, consistently. Finally, in the Maintenance stage, the aim is to sustain and avoid relapses. According to this model, behavioral change is often a complex and recursive process, and relapses to previous stages may occur before a new behavior is fully adopted (Prochaska and Velicer, 1997). For individuals to move from one stage to another, different mechanisms, and processes specific to the behavior in question, should be elicited. Some studies have implemented the Transtheoretical Model to understand the processes that female victims of IPV go through (Brown, 1997; Frasier et al., 2001; Burke et al., 2004). Additionally, research on health risk information has indicated the type of information that may trigger stage transition and ultimately affect behavioral change (Cismaru et al., 2008). Previous research has also applied the constructs and processes of the Transtheoretical Model in the context of domestic violence, revealing that it can be a valuable framework when considering the processes of change in victims leaving an abusive relationship, and in the treatment of perpetrators of violence (Levesque et al., 2008).

### Aims of the Present Study

Drawing from the literature on IPV directed at victimized men in DS/SS relationships and also from the theoretical models and conceptual frameworks pertaining to fear appeals and behavioral change, the present review research questions are:

• What are the general characteristics of existing pictorial IPV campaigns focusing on victimized men in DS/SS relationships? That is, to whom are they targeted, what type of approach do they use, what are their objectives and what type of information do they convey?
• Which of the main constructs of the EPPM, the Theory of Planned Behavior, the Elaboration Likelihood Model, and the Transtheoretical Model do these campaigns represent?

To the best of our knowledge, our study is the first to analyze these types of campaigns by characterizing their components according to a theoretically grounded framework. This approach is pertinent to inform future campaign developments because it allows tailoring campaigns to intended audiences by providing a framework by which their effectiveness can be assessed and potentially avoid undesired negative consequences.

## Materials and Methods

This review followed the ENTREQ statement, designed to promote and enhance transparent and comprehensive reports of synthesis in qualitative studies (Tong et al., 2012). The protocol for this study was previously registered at the PROSPERO–International Prospective Register of Systematic Reviews database (CRD42018115346).

### Searches

Searches were firstly conducted in the Web of Science, SCOPUS, PsycARTICLES, PsycINFO, Psychology and Behavioral Sciences Collection, Scielo, and B-On. In addition, governmental and non-governmental organization (NGO) websites were consulted. These sources allowed for the access to academic literature specific to the topic under research that has included pictorial campaigns focusing on victimized men. After concluding searches in these sources, free association searches were conducted in Google Images and gray literature. These specific searches allowed us to gather image files that fulfilled the inclusion criteria. The entire search process spanned 3 months—from January 2019 to March 2019.

#### Eligibility Criteria

National and international awareness pictorial campaigns focusing on victimized men in intimate relationships, in English, Spanish, and Portuguese, as still images (i.e., digital images, posters) released up until 2019. These languages were chosen based on their widespread use worldwide. These still images must have included (i) information and/or images relative to the formal promoting agency/entity; (ii) written text. Campaigns must have explicitly portrayed domestic violence or IPV concepts and topics, and campaigns about IPV forms (e.g., sexual abuse) were considered if explicitly stated in the campaign. Campaigns portraying other related topics (e.g., sexual assault) were also subject to analysis.

#### Exclusion Criteria

We excluded campaigns related to themes other than IPV (i.e., safe-sex, child abuse), promotion of products or brands, in languages other than English, Spanish, and Portuguese. Images that did not contain information (written text, images), relative to the formal promoting agency/entity, were also excluded. These criteria were defined to improve the focus of the campaign selection among images that were the product of creative/artistic work, and not the product of a formal agency/entity.

#### Keywords

Searches were carried out with different keywords using the Boolean operators “and” or “or” to articulate varied and specific results when possible. An example of search terms used is: (Campaign^∗^) AND (^∗^Violence) AND (Men). In what concerns searches in Google Images, different keywords were used such as “Campaign victimized men” and “Poster victim men” (see Supplementary Annex A for a comprehensive list).

### Record Screening

Using the PRISMA-P framework (Shamseer et al., 2015), a three-stage process of screening was implemented. One researcher screened all academic records by relevant keywords in the Title, then in the Abstract, and lastly a review of the body of the articles. Articles that did not mention any keywords either in the Title or Abstract were excluded. Regarding the images resulting from the Google Images searches, they were screened taking into consideration the inclusion and exclusion criteria and discussions with two other researchers that allowed for more transparency and congruence across the collected sample.

### Coding Taxonomy Development

The coding taxonomy was partially adapted from previous work on pictorial campaign characterization (Velez, 2014). On one hand, this taxonomy was deductively developed, considering the literature on IPV, the processes of seeking help among victims, and health communication design. On the other hand, its development was also inductive, grounded on the taxonomy application to a small sample of collected pictorial campaigns and to allow the emergence of additional codes that were meant to complement and improve the original coding scheme. The final version of our taxonomy was reached after conducting the coding of our entire sample. By applying the taxonomy to a wider range of campaigns, general codes were classified into specific subcategories to properly evaluate the content of the campaigns. By fine-tuning the coding scheme, new categories emerged that took into account unique aspects of our data. Thus, codes were organized to better represent the diversity of constructs in the campaigns, considering, when possible, a hierarchical progression in their specificity and distinctiveness from existing codes.

#### Taxonomy Categories

To adequately represent message content, different categories were created (see Supplementary Annex B for a summary). The first main group of categories represented the violence portrayed, perceived target audience, specific objectives, approach, and framing.

The second main group of categories was created considering the four theoretical models previously described and contemplated different constructs pertaining to each one of them. We considered the four main constructs of the EPPM and additionally Attitudes, Social Norms, and Behavioral Control surrounding IPV themes as stated in the Theory of Planned Behavior. Pertaining to the Elaboration Likelihood Model, categories mainly represented Dissonances, the person portrayed in the image (considered in this study as the message sources and a potentially important persuasive cue), and finally Colloquialisms. As for the different stages of change the campaigns may be useful for, we adapted a previously developed framework (Cismaru et al., 2008). According to this previously developed coding scheme, different threat appeal constructs (e.g., Self-Efficacy; Threat Severity) are useful for different stages (i.e., Contemplation and Preparation; Pre-Contemplation, Contemplation, and Maintenance, respectively). Different threat appeal constructs can target the same stage of change, given that through the behavioral change process, different types of information are important to highlight and they are often not mutually exclusive. Thus, using the results of the coding for the EPPM, we extrapolated for which stages the campaigns provided information for, considering specific categories for Pre-Contemplation, Contemplation, Preparation, Action, and Maintenance.

### Coding Process

Researchers used thematic analysis following a post-positivist paradigm (Braun and Clarke, 2006) to describe the content within the pictorial campaigns on a semantic level. Thematic analysis is a method for identifying, analyzing, and reporting patterns (themes) within data (Braun and Clarke, 2006). Based on a previously developed coding taxonomy, the majority of the content analysis was deductive (e.g., see Boyatzis, 1998). However, inductive codes emerged during the analysis, which were integrated into the taxonomy by establishing a clear definition of their meaning and their additional contribution to the analysis. The coders analyzed the content of the images and the text in each campaign and based on different examples for each category in the coding taxonomy, nominally coded it if it adequately represented that part of the content in the campaign (0 = absent; 1 = present). Most codes were not mutually exclusive, even if within the same category. This allowed for the most direct representation of the campaigns, considering that our coding taxonomy was developed posteriorly to the release of the campaigns under study, and we cannot assume that the constructs being analyzed anchored the campaigns’ development. Based on the agreement between the two coders regarding the fit of the coding scheme to the campaign analysis, a case-by-case analysis was employed to perform a semantic interpretation of the content. Given the novelty of our research approach to the topic, we decided to provide a balanced perspective between a rich thematic description of our entire data set and its most prevalent themes.

### Data Extraction

The practical characteristics of the campaigns, such as promoting entity, country of origin, year of release, and whether they were or not disseminated through multiple channels were collected. These data as well as the results of the coding process were registered in a Microsoft Excel form created for this purpose to simplify the inter-coder reliability index extrapolation. The promoting entities responsible for these campaigns were not contacted to clarify the intent behind their campaigns (only public information was considered).

### Risk of Bias (Quality) Assessment

A training session was conducted with the second independent coder to improve familiarity with the coding taxonomy. Then, one coder coded all pictorial campaigns, and the second coder coded a random selection of approximately 40% of these campaigns. IBM SPSS (v24) software for Windows 10, DAG_Stat tool (Mackinnon, 2000), and VassarStats (Lowry, 2019) were used to calculate the inter-coder reliability indexes [Cohen’s K, Prevalence and Bias Adjusted Kappa (PABAK), and Maximum Kappa (K_max_), when appropriate] indicating a Good to Very Good level of agreement according to the guidelines on inter-rater reliability levels (Nurjannah and Siwi, 2017) (see Supplementary Annex C for a detailed overview).

### Strategy for Data Synthesis

NVivo 12 software for Windows 10 was used. Results were aggregated in the form of overall frequency, taking into consideration that most codes within a category were not mutually exclusive. Phrases that were coded as colloquialisms were subjectively analyzed in the context of the language used and the information provided in the pictorial campaign. No formal coding process was involved in this analysis. Data synthesis was conducted by one researcher, with two others reviewing the process and providing feedback.

## Results

**FIGURE 1:**
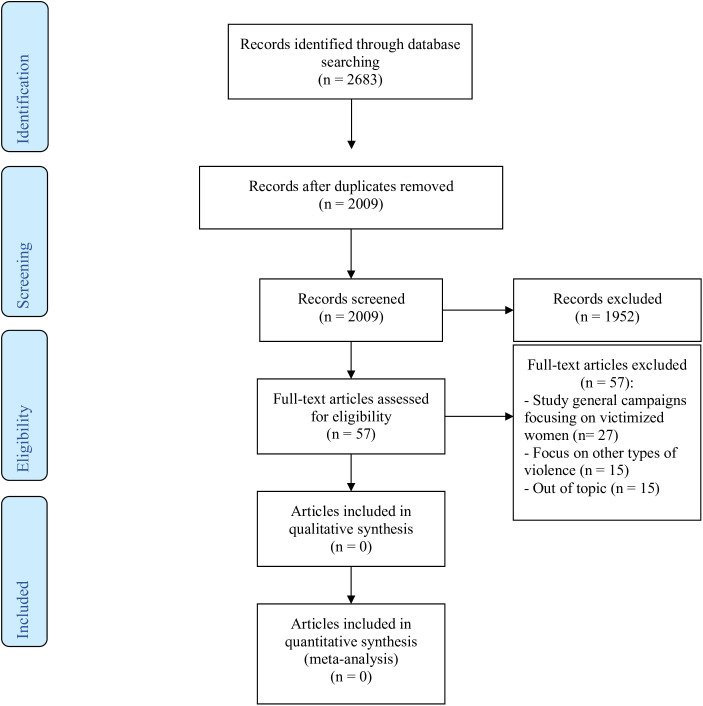
Flowchart of article search and screening process.

In total, 2,683 records were collected, and after duplicate removal, 2,009 records remained. After screening, only 57 were considered for full-text analysis. Based on the review process, no academic article collected studied campaigns specifically focusing on victimized men in DS/SS relationships nor featured images of a pictorial campaign (for an overview of the selection and screening process, see Figure 1). Given that no records resulted from the scientific database searches, additional searches were conducted in Google Images. This process followed a snowballing approach, identifying possible images *via* websites of NGOs and related images suitable according to the inclusion criteria.

A total of 134 images were collected. After an initial screening process, 28 were excluded for: (i) not referencing the promoting entity, (ii) portraying men exclusively as perpetrators, and (iii) not specifying violence toward men (ambivalent). Out of a total of 134 images, a short list of 97 images was screened again considering the eligibility criteria, with 40 images excluded for being: (i) developed as an individual’s art project, (ii) shared only on social media, and (iii) manipulated versions of existing images. Thus, a final sample of 57 images were considered for analysis, representing pictorial campaigns explicitly focusing on victimized men in DS/SS relationships, promoted by formal entities.

### Practical Characteristics of the Pictorial Campaigns

Concerning the campaigns’ practical characteristics, the release date ranged from 2004 to 2019. Twenty-three of these campaigns were disseminated using a multichannel approach. The majority of collected campaigns was released in England (*n* = 27) and was promoted mainly by NGOs and/or the police force (see Table 1 for a comprehensive overview). No data pertaining to the campaigns development or assessment were found online in the searches conducted.

**TABLE 1:** Campaign characteristics summary.

#	Promoting entity	Country	Year of release	Multichannel
1	ACON/LGBTIQ domestic violence interagency	Australia	2004	Yes
2	Virginia Sexual & Domestic Violence Action Alliance (The Red Flag Campaign)	United States	2006	No
3	Virginia Sexual & Domestic Violence Action Alliance (The Red Flag Campaign)	United States	2006	No
4	Virginia Sexual & Domestic Violence Action Alliance (The Red Flag Campaign)	United States	2006	No
5	Virginia Sexual & Domestic Violence Action Alliance (The Red Flag Campaign)	United States	2006	No
6	CIG	Portugal	2008	Yes
7	APAV	Portugal	2009	No
8	West Yorkshire Police	England	2010	No
9	Kirklees Council/West Yorkshire Police	England	2010	Yes
10	Kirklees Council/West Yorkshire Police	England	2010	Yes
11	The National Centre for Domestic Violence	England	2010	No
12	Safer Peterborough/White Ribbon	England	2013	No
13	NHS Forth Valley	Scotland	2013	No
14	Birmingham Safeguarding Adults Board	England	2013	Yes
15	End the Fear/Greater Manchester Police	England	2014	No
16	Kirklees Council	England	2014	Yes
17	End The Fear/Greater Manchester Police	England	2014	Yes
18	Derbyshire Police	England	2014	Yes
19	ACON/LGBTIQ Domestic Violence Interagency	Australia	2014	No
20	ACON/LGBTIQ Domestic Violence Interagency	Australia	2014	No
21	Kirklees Council/West Yorkshire Police	England	2014	Yes
22	Camden Safety Net	England	2014	Yes
23	Canadian Association for Equality	Canada	2015	No
24	Western Sydney Men And Relationships Services Interagency (WSMARS)	Australia	2015	No
25	Rainbow Bridge (Victim Support/Hate Report it Wales)	Wales	2015	No
26	Broken Rainbow/End the Fear	England	2015	No
27	Men Standing Up/Bradford Cyrenians	England	2015	Not available
28	LGBTQ partner abuse and sexual assault helpline (Virginia Department of Health/Virginia Sexual and Domestic Violence Alliance)	United States	2015	Yes
29	Association for Victim Support (APAV)	Portugal	2015	Yes
30	APAV	Portugal	2016	Yes
31	APAV	Portugal	2016	No
32	Surrey Police	England	2016	Yes
33	CiG	Portugal	2016	Yes
34	CIG	Portugal	2016	Yes
35	End the Fear/Greater Manchester Police	England	2016	Yes
36	Mankind Initiative	England	2016	Not available
37	Mankind Initiative	England	2016	Not available
38	Mankind Initiative	England	2016	Not available
39	United States Navy	United States	2017	No
40	APAV	Portugal	2017	No
41	APAV	Portugal	2017	No
42	COLEGAS-Confederación LGBT Española, Observatorio Español contra la LGBTfobia (StopLGBTfobia), Bufete Patón & Asociados	Spain	2017	Yes
43	Men’s Advice Line	Scotland	2017	Yes
44	Lancashire Victim Services	England	2017	Yes
45	Lancashire Victim Services	England	2017	Yes
46	APAV	Portugal	2018	Yes
47	Mankind Initiative	England	2018	Yes
48	West Yorkshire Police	England	2018	No
49	West Yorkshire Police	England	2018	No
50	Commission for Citizenship and Gender Equality	Portugal	2019	No
51	Men’s Sexual Health	England	Not available	No
52	White Ribbon	England	Not available	No
53	Abused Men in Scotland	Scotland	Not available	No
54	CPN Family Violence Program	United States	Not available	No
55	Abused Men in Scotland	Scotland	Not available	No
56	Sussex Police	England	Not available	No
57	Abused Men in Scotland	Scotland	Not available	No

### Global Characteristics of the Pictorial Campaigns

#### Perceived Campaign Main Target

Several of the selected campaigns only stated that men suffer violence in their relationships, not defining the type of relationship they were in (*n* = 22, 39%). They were followed by campaigns focusing on victimized men in SS relationships (*n* = 21, 37%) and DS relationships (*n* = 20, 35%). Eight of these campaigns focusing on victimized men in DS/SS relationships explicitly stated that violence existed both in SS and DS relationships. Among campaigns targeting bystanders, 10 were specifically directed at friends of the victim (18%), while four were not specifically aimed at bystanders in particular but for general bystanders (7%). Finally, out of the selected 57 campaigns, only one targeted female perpetrators (2%) and three (5%) did not define who the perpetrator was.

#### Perceived Main Objectives

The vast majority highlighted the problematic nature of domestic violence/IPV and its consequences (*n* = 48, 84%), while introducing new and specific useful information to recognize the problem and escape violence (*n* = 46, 81%). As for the overall approach, most campaigns included an interventive approach (*n* = 54, 95%), followed by prevention (*n* = 21, 37%), and finally post-violence or treatment (*n* = 9, 16%).

##### Information provided

Concerning the informative component, most campaigns (*n* = 46, 81%) highlighted that violence toward men in intimate relationships is a serious and real problem. Thirty-five campaigns specifically represented psychological violence (61%), 26 mentioned the social repercussions of the violence (46%), and 22 informed about the physical consequences of violence (39%). Three campaigns identified economic manipulation as a form of violence (5%), and only one campaign presented information about the legal rights and protection of the victim (2%).

Some campaigns also highlighted the emotional struggles that victims often face. Twenty-nine campaigns (51%) emphasized that men in these problematic situations suffer in silence and often feel powerless to act (e.g., “As a man, telling somebody that your partner is abusing you is difficult. You might feel ashamed, embarrassed, or worried you’ll be viewed as less of a man”–#11). Another 12 campaigns (21%) stated that violence often occurs in privacy and is not publicly displayed (e.g., “Shaun’s familiar with control and isolation… That’s because he’s not allowed to see his family or friends”–#55). Four campaigns (7%) highlighted the specific additional stressors in an IPV context to gay, bisexual, trans, and intersex men (e.g., “I’ll out you”–#25). Finally, regarding formal support networks, 47 campaigns (82%) informed of formal support mechanisms such as hotlines and websites (e.g., “Call the Men’s Advice Line and talk it over”–#43).

### Theoretical Representation in the Campaigns

#### The Extended Parallel Processing Model

In our sample, the majority of campaigns (*n* = 53, 93%) displayed a recommended response (e.g., hotline, website). However, only 20 of these 53 campaigns (38%) informed about the efficacy of the recommended response as stated in the model (e.g., “A confidential and specialized LGBT domestic abuse service centered around YOU and YOUR needs in Cardiff”–#25). Most campaigns (*n* = 46, 80%) also highlighted the severity of the threat (e.g., “Women and men affected by domestic violence can lose their confidence, feel mentally defeated, and become depressed and very anxious”–#10), and out of these 46 campaigns, 16 campaigns (35%) visually represented bruises, cuts, and expressions of suffering on the victim. The remaining 30 campaigns (65%) framed violence implicitly with text, offering information on severity nonetheless. When considering our entire sample, messages that promoted the viewer’s perception of self-efficacy were moderately frequent (*n* = 32, 56%; e.g., “You deserve respect, not pressure”–#28). Information regarding susceptibility to the threat portrayed in the campaigns was the least common of all constructs (*n* = 13, 23%; e.g., “1 in 4 LGB and 3 in 4 Trans men and women will experience some form of domestic abuse in their lifetime.”–#35). When considering the entire sample and the recommendations regarding fear appeals, only four (7%) campaigns represented all four constructs of the EPPM simultaneously. When analyzing the most prevalent combinations of three of this model constructs, only six (11%) represented the efficacy of the recommended response, severity of the threat, and self-efficacy-inducing information.

#### Theory of Planned Behavior

Our analysis indicated that most campaigns aimed to generate behavior change (*n* = 51, 89%; e.g., “Break the silence, report it”–#51). Subsequently, they aimed to change attitudes and beliefs (*n* = 47, 82%; e.g., “For all victims of domestic abuse, the advice is the same, you are not alone and there is help available”–#11). Thirteen (23%) contrasted the importance of the victims’ well-being with currently biased social norms that could indicate otherwise (e.g., “Derek makes sure the neighbors don’t hear the smashing… that’s because who’d believe that he was on the receiving end?”–#57). Finally, another seven (12%) highlighted the importance of changing gender norms (e.g., “Because men are traditionally thought to be physically stronger than women, you might be less likely to talk about or report abuse”–#54). All these analyses pertaining to social norms were simultaneously coded in the campaigns eliciting behavior and attitude change. Finally, 14 (25%) campaigns integrated constructs representing behavioral control, attitudes, and norms related to IPV.

#### Elaboration Likelihood Model

Our findings highlight the representation of dissonances, the properties of the perceived message source, and of the perceived victim of violence in the campaigns.

##### Dissonances

We found that dissonances were used to represent facilitators to the dissolution of violence, such as highlighting the undesired state the victim is in when viewing the campaign and highlighting a new and more healthy state that he could achieve if he seeks help. In many campaigns (*n* = 41, 72%), portraits of the victim’s powerlessness contrasted with a message empowering the victim to act (e.g., “Break the silence. Break the cycle. Domestic Abuse. We know it happens. Contact us. We can help you.”–#18). Other campaigns highlighted that what was culturally known about violence in intimate relationships was a limited perspective on the subject, subsequently presenting the viewer with up-to-date and more complete knowledge about the nature of violence and its expressions and consequences (*n* = 30, 53%; “Being trapped in a relationship by someone else’s insecurities is domestic abuse. If this is you or someone you know, get confidential help now.”–#21). A smaller number of campaigns contrasted love and abuse, explaining their incompatibility (*n* = 12, 21%; “She puts me down every chance she gets. Know this isn’t love.”–#22), and six (11%) highlighted that the victim status was not only applicable to women and that violence can affect any gender (i.e., “It’s not only women that can be abused by their partner”–#37). Regarding the use of colloquialisms, 27 (47%) campaigns presented some form of culturally specific term or expression that could potentially facilitate persuasion (i.e., “Does that sit right with you”–#35; “Stand up”–#19; “Red Flag”–#4).

##### Perceived message source

In our analysis, we considered the person portrayed in the campaign as the perceived message source. Pertaining to the type of relationship the victim was in as represented by the information provided in the campaigns, we found that 15 campaigns (26%) did not present any details that could inform on the type of relationship or of his sexual orientation and/or gender identity. Furthermore, it was found that the same number of campaigns explicitly informed that the victim was in a DS relationship (*n* = 8, 14%) or in an SS relationship (*n* = 8, 14%).

##### Perceived victim

On a different level of analysis, our findings revealed that the victim was displayed by himself in 35 campaigns (61%), with six additional campaigns portraying both the victim and the perpetrator simultaneously (11%). When the victim was presented alone, the majority (*n* = 22, 63%) represented the victim suffering, followed by a more intimate portrayal in which the victim’s face was directed at the campaign viewer (*n* = 18, 51%). When considering the six campaigns that presented both the victim and the perpetrator simultaneously, three campaigns represented reenactments of acts of violence (50%). Considering all the campaigns that portrayed victims, 26 portrayed what we perceived as young Caucasian males (65%), followed by middle-aged Caucasian men (*n* = 6, 15%). Representing Black men as the victims of IPV was significantly less prevalent, being present in only two campaigns (5%).

#### The Transtheoretical Model

By considering the threat information that was present in our sample of campaigns, we extrapolated the stages of change the campaigns could be useful for using an adaptation of a previously developed coding scheme (Cismaru et al., 2008) (see Table 2 for an overview). We found that the threat information represented in the campaigns was mostly directed at targets in the Maintenance stage (*n* = 52, 92%) and Contemplation stage (*n* = 51, 89%). These were followed by the Pre-Contemplation (*n* = 50, 88%) and Preparation (*n* = 39, 68%) stages. Lastly, information that was useful for the Action stage was the least prevalent in our sample (*n* = 32, 56%), being present nevertheless in more than half of the campaigns. When analyzing the co-occurrence of codes pertaining to the stages of change, we found that 28 (49%) campaigns presented threat information that was adequate for all five stages of change simultaneously. Finally, only three campaigns (5%) did not provide any information for any stage of change given the absence of threat components.

**TABLE 2:** Extended Parallel Processing Model and Transtheoretical Model coding distribution.

		Extended parallel processing model			
		
Transtheoretical Model	# Campaigns	Threat severity	Threat susceptibility	Response efficacy	Self-efficacy
Pre-contemplation	49	46	13	17	29
Contemplation	51	45	12	19	32
Preparation	39	31	9	21	32
Action	32	26	8	15	31
Maintenance	52	46	13	19	32

## Discussion

The first aim of the present study was to identify the characteristics of existing pictorial IPV campaigns focusing on victimized men in DS/SS relationships.

We start by highlighting the relative recency of these initiatives in the IPV field, given that the earliest released campaign was launched in 2004. Additionally, in our selected sample, England was the country with the highest number of campaigns. Although there have been many efforts to mitigate IPV in the past (World Health Organization [WHO] and London School of Hygiene and Tropical Medicine, 2010), the societal, legal, and clinical recognition of men as victims of IPV in their intimate relationships is a relatively recent topic. The lack of visibility on the topic may have contributed to the reduced number of campaigns, but on the other hand, the lack of campaigns may have also played a role in the invisibility of this topic and victimized men in society. Thus, an increase in the number and quality of future campaigns focusing on victimized men may play a positive role in the social recognition of this issue. Additionally, given that our systematic search of the literature did not return any studies on this specific topic, to the best of our knowledge, the present study is the first to describe and analyze campaigns focusing on men as victims of IPV, and specifically pictorial campaigns.

Secondly, our study examined the campaigns’ main targets, their main objectives, as well as their informative components to contribute to a better characterization and understanding of the target populations on which they aimed to intervene.

We found that the majority of campaigns only mentioned that men suffer violence in their relationships but did not define what specific relationship types they were in. The importance of tailoring campaigns to the specific social and psychological profiles of the target audience has been highlighted previously (Maibach and Cotton, 1995; Palmgreen et al., 1995), but our findings suggest that most campaigns do not do so. For instance, GBTI men may not see themselves in the ambivalent campaigns due to heteronormativity. It is nevertheless very positive that a relatively high number of campaigns has focused on victimized men in SS relationships, suggesting that campaign developers may be more aware and inclusive. Additionally, campaigns focusing on bystanders may play a crucial role in increasing the recognition of violent attitudes and behaviors, as well as leading the victim of violence to act (Potter et al., 2011). We did not find information on the formative research processes of these campaigns and their objectives, but they have the potential to model prosocial bystander behaviors (Potter et al., 2009).

Most campaigns in our sample aimed to intervene, which may reflect an adequate strategy to urgently help those who are in need. Nevertheless, fewer campaigns focused on information that can be preventive in nature. In the future, this type of information could be highlighted more often given that it may represent the first step in the recognition of violence in the viewer or those surrounding him. Additionally, future campaigns could emphasize that no matter the approach, psychological help is available and is a crucial step in the entire process (Machado et al., 2016).

Furthermore, many campaigns portrayed the different expressions the violence can assume, representing mainly psychological, social, and physical violence. This information is essential to better clarify what exactly constitutes violence and how often it emerges in abusive relationships. Nevertheless, there is still a lack of focus on less commonly discussed types of violence, such as sexual abuse and economic manipulation. Furthermore, it is important to consider the specific aggressions GBTI men may face, such as threatening to out the victim as GBTI or as HIV positive in the workplace or family context (Edwards et al., 2015b). Finally, the campaigns’ emphasis on the silence and sense of powerlessness of the victims is a frequent topic in the literature surrounding victimized men (Cook, 2009; Edwards et al., 2015b; Machado et al., 2016) and may adequately portray the feelings these men have. This information may help men with underlying feelings of shame and stigma surrounding their own recognition as victims, as well as seeking help.

For our second research question, we aimed to understand how content in the campaigns could be representative of constructs of the different theoretical models.

Pertaining to the EPPM (Witte et al., 2001), we only found four campaigns that thoroughly followed the recommendations of the model on threat appeal design. Subsequently, 12 others presented three of the constructs simultaneously. These findings point to a need for campaign developers to adequately frame message information to consider these threat components when suitable, so that the message can be processed and not denied or avoided (Witte et al., 2001). Inadequately framing threatening appeals may reinforce undesired attitudes and behaviors (i.e., Boomerang Effect; Witte et al., 2001), possibly harming victimized men indirectly. Intrinsically linked with the threat severity information presented, we verified that the majority of campaigns framed violence implicitly and thus did not visually represent bruises and cuts on victimized men. This is an aspect to be highlighted, given that portraying men suffering significantly may be harmful for victimized men. Considering victimized women’s mostly negative impressions on IPV campaigns targeted at them (West, 2013), conducting formative research with the target population may help to understand what type of information is able to induce high perceived threat severity. Nevertheless, it may be possible that using an explicit approach can be useful for a given population. Information about threat susceptibility was the least frequent of all in our sample, contrasting with its high relevance for preventive approaches. Additionally, despite providing many different recommended responses, information about their efficacy was significantly less available. This may impact the facilitation of attitudes and behaviors toward the recommended response and ultimately drive men not to act. Information on how the support system works and how it is effective seems to be crucial information in breaking men’s negative expectations about how they will be treated when they act to escape the violence (Machado et al., 2016). This motivation to act is linked with the viewer’s own sense of self-efficacy, and in our sample, information on this component was moderately frequent. Future campaigns will likely benefit from eliciting this component in the victim given that previous research has suggested that it is crucial for beneficial attitude and behavior change in the IPV context (Cismaru and Lavack, 2010; Overstreet and Quinn, 2013; Edwards et al., 2015a).

Pertaining to the Theory of Planned Behavior, we found that the emphasis on generating behavior, attitude, and belief change was very positive and adequately served the perceived general purposes of the campaigns under study: to inform the viewer and lead the person to act. Despite this, the lack of focus on changing social norms surrounding violence, gender, and help-seeking represented an aspect that needs improvement. The victims’ perception of what these specific social norms are is key to trigger actual behavioral intention, and previous literature has highlighted the pressure exerted by social expectations that is felt by victimized men (Cook, 2009; Machado et al., 2016). Furthermore, campaign developers might consider if the perceived norms should be mitigated, changed, or reinforced, and when so, specify exactly what those norms pertain to (Dillard and Pfau, 2002). Nevertheless, in our sample, a small number of campaigns portrayed all three constructs of the Theory of Planned Behavior. Despite the possible positive increment added to the message, future research could test if all the aforementioned constructs in a message are relevant for eliciting any given behavior change.

Regarding the Elaboration Likelihood Model and its constructs, the use of dissonances was widespread, and we found that the information presented to the viewer was in accordance with the literature on how victims feel when they are targets of IPV (Cook, 2009; Machado et al., 2016). For example, contrasting a sense of powerlessness with a specific indication to act may reflect a careful consideration of the campaign developers about what the victim may be feeling and what could potentially elicit change. This can indirectly empower men to act, but if no information is provided about self-efficacy or how the recommended action is effective, men may feel overwhelmed and still not ready to act. Dissonances about social norms and the nature of violence are also key parts of these campaigns, as they can provide a new perspective on a topic that for these men may have seemed unsurmountable or even not recognizing it as an issue at all. The use of colloquialisms is a positive increment, but according to the literature, they must be used only when they have been pretested with the target population (Witte et al., 2001). Not pretesting a certain colloquialism may lead to unintended reactions and wrongful appropriations of the term in use, something that could counter the desired effect of the message. Finally, in what concerns the message source, more than half of the campaigns visually portrayed a victim, and among these, more than half represented the victim suffering. As stated before, caution should be taken when considering this aspect of campaigns. When considering who could be portrayed in future campaigns, other sources could be tested as previous literature on the role of message sources did not find that the use of role models made a significant contribution to effect size under study (Hornik, 2002). In the absence of a role model, specific characteristics of the source are relevant for consideration, such as credibility (Hass, 1981) and attractiveness (Chaiken, 1979). In line with these characteristics, it could be possible that including a real testimonial from an empowered male victim could be beneficial for victimized men. Furthermore, previous literature has indicated that IPV perpetration and victimization are transversal to a diversity of sociodemographic profiles (CDC, 2010). Thus, more diversity should be considered in the victims who are portrayed, so that they could represent male victims of different ethnicities, age groups, economic status, sexual orientations, and degree of functionality.

Pertaining to our findings on the Transtheoretical Model, it is positive that many campaigns simultaneously provide information that is useful for any stage of change. Nevertheless, in accordance with the principles of formative research, the main stages of change in the target population should be assessed before development is initiated (Maibach and Cotton, 1995; Noar, 2006). In line with this perspective, campaign developers must make sure that at least the stages of readiness in which the target population is are considered in the information provided by the campaign.

In summary, to surmount the limitations in our sample, our results suggest that the development of future campaigns could consider the core constructs and processes underlying behavioral change, fear appeals, and persuasion. Overall, public information could be made available regarding the development of campaigns and how they were tailored to the target population. Grounding this development on theoretical approaches provides a comprehensive framework on which the characteristics of an audience and the performance of a campaign can be analyzed. This framework facilitates a structured analysis of a campaign’s effectiveness (or lack thereof), eventually unintended effects, and how these could potentially be overcome. This approach may, in turn, benefit changes in the target population while reducing costs that often occur with trial-and-error approaches.

Finally, for the analysis of future campaigns in this field or others that may apply, the taxonomy in use for our content analysis may be useful as it provides a theoretically grounded approach. Despite being developed specifically for the field of IPV (both directed at self-identified men and women), and despite considering the literature on IPV, campaign design, threat appeal research, and LGBTI topics, it may be improved in the future to consider other types of media, such as video campaigns.

### Limitations

To the best of our knowledge, this is the first study to assess campaigns directed at victimized men, and as such it used qualitative methods to extract crucial data for its understanding. Considering preset outcomes and effectiveness indicators, future studies could use quantitative methods to assess the perceived effectiveness of these campaigns and how different constructs play a role in leading the person to be more aware, to change the person’s attitudes, and even to leave their abusive relationships.

It is also important to consider that each individual has a limited cognitive capacity to process all stimuli adequately, and thus the processing of each of these campaigns may differ with other viewers (Lavie, 2005). Nevertheless, such a fact highlights the importance of using the Elaboration Likelihood Model as a framework to study these campaigns, as peripheral cues can capture and hold our attention and lead to persuasion.

Another important consideration is that due to the definition of codes in our coding taxonomy, many of the codes were not mutually exclusive. This allowed us to extract the properties of the campaigns as best as possible, but also highlight the limitations of the taxonomy that realistically could not accommodate all variations of the codes in an image.

Additionally, there are other theoretical models that could be suitable to proceed with the analysis, such as the Protection Motivation Theory by Rogers (1975) or the Health Belief Model (Rosenstock, 1974). Other campaigns targeting victimized men could be available in other channels such as TV and radio and in other languages besides English, Spanish, and Portuguese.

Finally, we did not consult with campaign developers, and thus our extrapolations were achieved with the information provided by the campaign. Future campaigns could provide more public information about their development and assessment, including pretest–posttest designs and longitudinal studies that assessed attitude and behavior change over time.

## Conclusion

The present study aimed to understand how IPV pictorial campaigns focusing on victimized men in DS/SS relationships were characterized, and how they represented constructs of the EPPM, Theory of Planned Behavior, Elaboration Likelihood Model, and Transtheoretical Model. Our findings revealed that, despite being relatively recent, many of the campaigns integrated key constructs of these theoretical models and were at least partially in line with literature recommendations on the design of fear appeals and the EPPM. A great focus was given to the elicitation of behavior and attitude change, as well as the use of peripheral cues that could capture the viewer’s attention. Nonetheless, more information could be present on how social norms condition the lives of victimized men and their attempts to seek help, as well as how susceptible men are to the violence. In the future, public campaigns could provide more data on their development and effectiveness in order to improve current knowledge on campaign design and its effectiveness. Finally, more quantitative research is needed to truly understand the effects that different constructs portrayed in these campaigns have on the viewers.

## Data Availability Statement

The datasets generated for this study are available on request to the corresponding author.

## Author Contributions

ER, PA, and CM contributed to the conception and design of the present study, with ER specifically conducting the literature review, and developed the Supplementary Material. ER, PA, CM, and XH idealized and revised the methodological approach. ER carried out data collection, but all authors contributed to its analysis and later the development and revision of the discussion of the findings. All authors contributed to manuscript revision and read and approved the submitted version.

## Conflict of Interest

The authors declare that the research was conducted in the absence of any commercial or financial relationships that could be construed as a potential conflict of interest.
