# Corrigendum: Pictorial Campaigns on Intimate Partner Violence Focusing on Victimized Men: A Systematic Content Analysis

**DOI:** 10.3389/fpsyg.2020.588807

**Published:** 2020-10-22

**Authors:** Eduardo Reis, Patrícia Arriaga, Carla Moleiro, Xavier Hospital

**Affiliations:** ^1^Social and Organizational Psychology Department, CIS-IUL/ISCTE-Instituto Universitário de Lisboa, Lisbon, Portugal; ^2^Inclusive Policy Lab, UNESCO, Dakar, Senegal

**Keywords:** intimate partner violence, victimized men, pictorial campaigns, prevention, help-seeking

In the original article, there was a mistake in Figure 1 as published. In the section that lists the number of full-text articles excluded and the reasons for exclusion, 15 articles listed as “Out of topic” were missing. By including these 15 articles, the total amount of articles excluded adds up to the stated 57. The corrected Figure 1 appears below.

**Figure 1:**
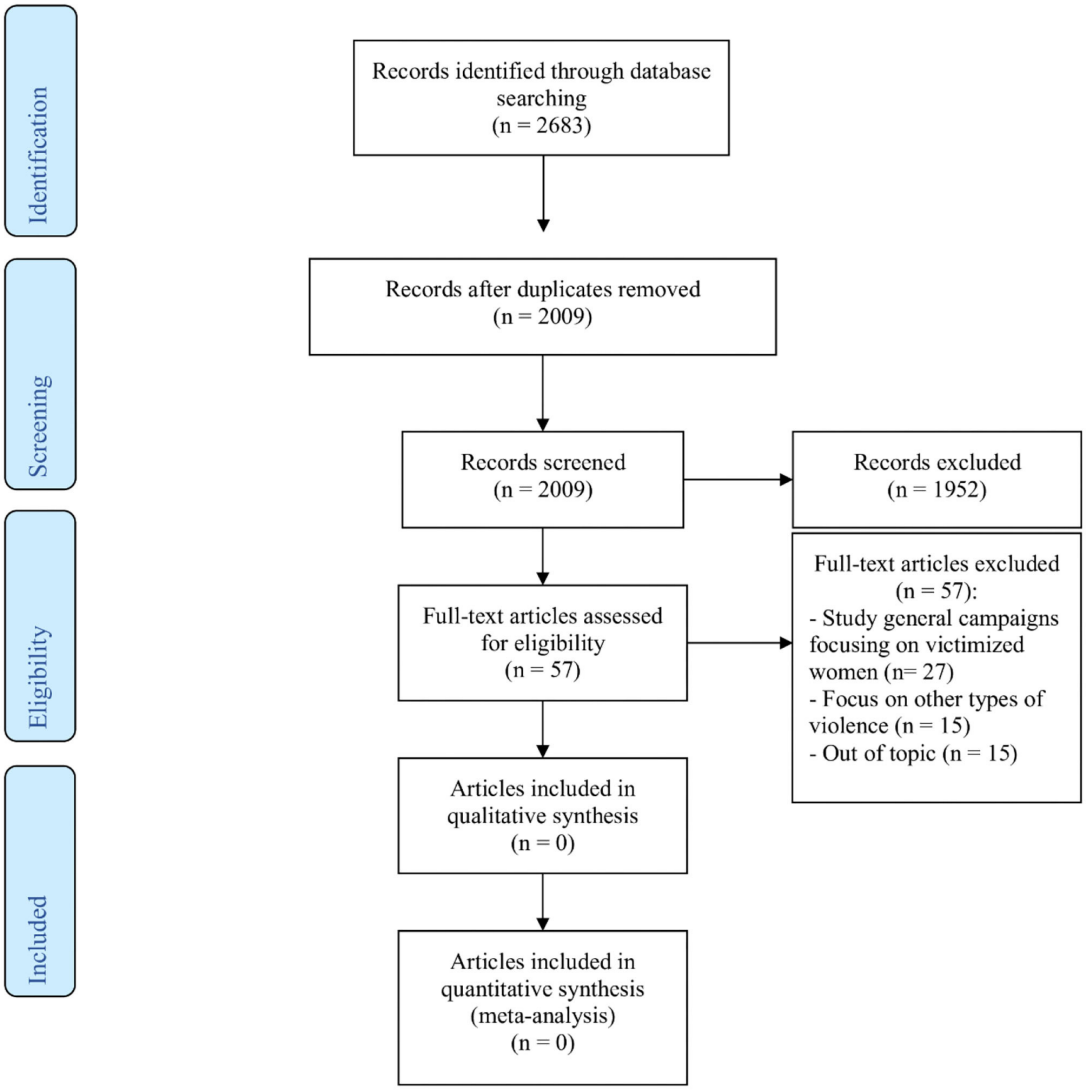
Flowchart of article search and screening process.

The authors apologize for this error and state that this does not change the scientific conclusions of the article in any way. The original article has been updated.

