# Supplementary material for: Pictorial Campaigns on Intimate Partner Violence Focusing on Victimized Men: A Systematic Content Analysis
Source: Front Psychol. 2020 Jul 9;11:1450. doi: 10.3389/fpsyg.2020.01450 (PMC7363929; doi:10.3389/fpsyg.2020.01450)
Supplement: Supplementary file 1 [file Table_1.DOCX]

Annex A. Detailed overview of search terms and boolean operators

| Language | Search Terms |
| --- | --- |
| English | (Campaign*) OR (Prevention) OR (Poster) AND (*Violence) OR (IPV) OR (*Abuse) OR (Victim) AND (Men) OR (LGBT) OR (Gay) OR (Bisexual) OR (Transgender) OR (Heterosexual) |
| Spanish | (Campaña*) OR (Prevención) OR (Poster) AND (*Violencia) OR (VRI) OR (*Abuso) OR (Víctima) AND (Hombre) OR (LGBT) OR (Gay) OR (Bisexual) OR (Transgenero) OR (Heterosexual) |
| Portuguese | (Campanha*) OR (Prevenção) OR (Poster) AND (*Violência) OR (VRI) OR (*Abuso) OR (Vítima) AND (Homem) OR (LGBT) OR (Gay) OR (Bissexual) OR (Transgénero) OR (Heterossexual) |

Annex B. Coding Taxonomy (adapted from Velez, 2014)

| Theme | Category | Subcategory | (sub) Subcategory |
| --- | --- | --- | --- |
| Perceived main target of the message | Victimized man/men | Different-sex relationship |  |
|  |  | Same-sex relationship |  |
|  |  | Relationship type / gender identity / sexual orientation not specified |  |
|  | Perpetrator(s) | Female |  |
|  |  | Male |  |
|  |  | Gender identity not specified |  |
|  | Bystander(s) | Friends |  |
|  |  | Family |  |
|  |  | Work colleagues |  |
|  |  | Public witnesses |  |
|  |  | Not specified |  |
|  | Institutional Agents | Police Forces |  |
|  |  | Health Professionals |  |
|  |  | Schools/Universities |  |
|  |  | Policy-Makers |  |
|  |  | Not specified |  |
|  | General Population | Teenagers |  |
|  |  | Older adults |  |
|  |  | Females |  |
|  |  | Males |  |
|  |  | Not specified |  |
|  | LGBTI Population | Teenagers |  |
|  |  | Older adults |  |
|  |  | Females |  |
|  |  | Males |  |
|  |  | Trans and Non-binary people |  |
|  |  | Not specified |  |
| Perceived message objective (adapted from Buunk & Van Vugt, 2008) | Remind of the existing problem (i.e. bring awareness to the existing problem) |  |  |
|  | Provide new knowledge (i.e. provide articulate information regarding the nature, processes and consequences of IPV, as well as inform of available resources) |  |  |
| Approach (Rappaport & Seidman, 2000) | Prevention (i.e. encourages the population to consider their behavior and intervene preemptively) |  |  |
|  | Intervention (i.e. aimed at men at risk, highlighting the recognition of the need for help, and the use of formal resources available before violence escalates further) |  |  |
|  | Treatment / Post-violence (directed at men and/or their families/friends after abuse has occurred, suggesting support and treatment if necessary) |  |  |
| Method (adapted from Buunk & Van Vugt, 2008) | Descriptive information | About the existence of violence directed at men (e.g. statistics) |  |
|  |  | About the particularities of violence directed at GBTI men (e.g. threatening to “out” the partner) |  |
|  |  | About the privacy of the phenomenon (i.e. occurrence of violence mostly in the private space of a home) |  |
|  |  | About the silence/powerlessness of the victims (e.g. men can stay silent for years) |  |
|  |  | About the consequences of violence for the victim | Physical (e.g. wounds, cuts) |
|  |  |  | Psychological (e.g. insults, manipulation) |
|  |  |  | Sexual (e.g. rape) |
|  |  |  | Social (e.g. forced social exclusion) |
|  |  |  | Economic (e.g. manipulation of funds by the perpetrator) |
|  |  |  | Legal (e.g biases towards the perpetrators in the legal system) |
|  |  |  | Moral (e.g. going against the victim’s moral principles) |
|  |  | About the formal mechanisms for help and support (e.g. hotlines, NGO’s) |  |
|  | Valence of the appeal (Henley, Donovan & Moorhead, 1998, Paek, Kim & Hove, 2010) | Negative (i.e. elicits negative emotions and sensations, is unpleasant) |  |
|  |  | Neutral (i.e. does not elicit any relevant sensations or emotions, is neutral) |  |
|  |  | Positive (i.e. elicits positive emotions and sensations, is pleasant) |  |
| Extended Parallel Processing Model (Witte, Meyer & Martell, 2001) | Threat Severity (i.e. The magnitude of harm expected from a threat. The significance or seriousness of a threat. The degree of physical, psychological, or economic harm that can occur.) | Framing of the Violence | Explicit (i.e. images of victimized men with wounds, bruises and other physical repercussions) |
|  |  |  | Implicit (i.e. identifying that violence occurs to men in general, no visual representation of violence and/or its consequences on the victims) |
|  | Threat Susceptibility (i.e. The likelihood that a specific person or audience will experience a threat. The degree of vulnerability, personal relevance, or risk of experiencing a threat.) |  |  |
|  | Self-Efficacy (i.e. The degree to which the audience perceives they are able to perform the recommended response to avert the threat. Sometimes self-efficacy is called "efficacy expectations", and answers the question "what do you expect will happen if you attempt to perform a certain behavior?") |  |  |
|  | Efficacy of the Recommended Response (i.e. The degree to which the recommended response effectively averts the threat. Sometimes it is called "outcome expectations", and answers the question "if you perform a certain behavior, what do you expect the outcome to be?") |  |  |
| Theory of Planned Behavior (Ajzen, 1991) | Generate attitude and belief change (i.e. change the evaluations that viewers make on determined questions and topics related to IPV) |  |  |
|  | Generate behavior change (i.e. change behaviors, and in this sense, elicit help-seeking, information gathering, and/or other related topics in IPV processes such as leaving the abusive relationship) |  |  |
|  | Need for change in gender roles (i.e. information that elicits critical thinking regarding what men and women are traditionally thought to be and how to behave; aims to question different forms of conditioning that may hamper the well-being of men in IPV situations) |  |  |
|  | Highlight the importance of well-being despite social norms (i.e. reinforce that despite any given social context and its associated norms regarding, the well-being of the victim is what matters most; confronts topics such as guilt and shame to report the victimization) |  |  |
| Elaboration Likelihood Model (Petty & Cacioppo, 1986) | Dissonances that facilitate the dissolution of violence (i.e. highlighting the contrast between the state the victim is in with the state that he could be in if a given attitude or action is adopted; uses a gain frame approach) | Previously biased notions about violence VS up-to-date and correct information provided by the campaign |  |
|  |  | Victim status applied only to women VS violence is genderless |  |
|  |  | Love VS abuse |  |
|  |  | Powerlessness of the victim to act VS empowerment of the victim to act |  |
|  | Colloquialisms (i.e. Informal reference used to describe something with an attached symbolism or meaning within a certain cultural group) |  |  |
|  | Violence Portrayed | Masculine gender-expression victim | Different-sex relationship |
|  |  |  | Same-sex relationship |
|  |  |  | Relationship type / gender identity / sexual orientation not specified |
|  |  | Perpetrator | Person with feminine gender expression |
|  |  |  | Person with masculine gender expression |
|  |  |  | Person with gender expression not specified |
|  | People represented in the campaign | Just the victim |  |
|  |  | Just the perpetrator |  |
|  |  | Victim and perpetrator |  |
|  | Types of emotions/situation represented on the people present in the campaign | Demonstrations of suffering (e.g. crying, laying head down) |  |
|  |  | Intimate (e.g. close-ups) |  |
|  |  | Reenactment of acts of violence (e.g. slap on the face, use of knife aimed at victim) |  |
| Transtheoretical Model (Prochaska & Velicer, 1997; adapted from Cismaru & Lavack, 2010) | Pre-Contemplation (i.e. susceptibility and severity to the threat should be increased by encouraging people to recognize the signs of an abusive relationship and the consequences of being in an abusive relationship.) |  |  |
|  | Contemplation (i.e. campaigns should frame change as beneficial and increase self-efficacy, while highlighting the severity of being in an abusive relationship and be given confidence in their ability to make changes (higher self-efficacy.) |  |  |
|  | Preparation (i.e. messages should communicate that people can improve their lives and that there are significant benefits in doing so. People should be told to accept the inevitable anxiety that accompanies the recognition that they might fail with their efforts. Planning small steps leading to a course of action that prepares for behavioral change is recommended) |  |  |
|  | Action (i.e. campaigns should increase the perceived benefits associated with ending domestic violence situations. They should also increase confidence in performing the recommended behaviors, as well as overcoming the discomfort associated with change. Campaigns should also encourage people to commit to ending domestic violence.) |  |  |
|  | Maintenance (i.e. Campaigns should focus on making people feel rewarded for sustained behavior. They should be reminded of the dangers associated with their former behavior.) |  |  |

Annex C. Detailed overview of interrater reliability information for each code

| Code Definition | Cohen’s Kappa | (Lower 95% CI,  Upper 95% CI) | Observed Agreement | Chance Agreement | Positive Agreement | Negative Agreement | Prevalence & Bias Adjusted Kappa | Byrt’s Prevalence Index (PI) | Byrt's Bias Index (BI) | K_max_ |
| --- | --- | --- | --- | --- | --- | --- | --- | --- | --- | --- |
| Campaign aimed at men in different sex relationships | 1 | 1 | 1 | 0,5918 | 1 | 1 | 1 | -0,4286 | 0 | 1 |
| Campaign aimed at men in same-sex relationships | 0,774 | (,3534 - 1,1949) | 0,9524 | 0,7891 | 0,8 | 0,973 | 0,9048 | -0,7619 | -0,0476 | 0,7742 |
| Campaign aimed at men in relationships (not defined / no information) | 0,901 | (0,7137 - 1,0891) | 0,9524 | 0,517 | 0,96 | 0,9412 | 0,9048 | 0,1905 | 0,0476 | 0,9014 |
| Campaign addressed to bystanders (Friends) | 0,644 | (0,0079 - 1,2803) | 0,9524 | 0,8662 | 0,6667 | 0,9744 | 0,9048 | -0,8571 | -0,0476 | 0,6441 |
| Campaign addressed to bystanders (not defined) | 0,351 | (-0,1669 - 0,8679) | 0,8571 | 0,78 | 0,4 | 0,9189 | 0,7143 | -0,7619 | -0,1429 | 0,3505 |
| Campaign addressed to female perpetrators | 0,0000 | 0,0000 | 0,9524 | 0,9524 | 0 | 0,9756 | 0,9048 | -0,4524 | -0,0476 | 0,0000 |
| Campaign aimed at perpetrators (not defined) | 0,7742 | (0,3534 - 1,1949) | 0,9524 | 0,7891 | 0,8000 | 0,9730 | 0,9048 | -0,7619 | -0,0476 | 0,7742 |
| Objective: To remind of the existing problem | -0,0500 | (-,01192 - ,0192) | 0,9048 | 0,9093 | 0,9500 | 0,0000 | 0,8095 | 0,9048 | 0,0000 | N/A |
| Objective: to introduce new knowledge | 0,7410 | (,4107 - 1,0708) | 0,9048 | 0,6327 | 0,9375 | 0,8000 | 0,8095 | 0,5238 | 0,0952 | 0,7407 |
| Approach: Prevention | 0,7123 | (,4124 - 1,0122) | 0,8751 | 0,5034 | 0,8421 | 0,8696 | 0,7143 | -0,0952 | -0,0476 | 1,0000 |
| Approach: Intervention | -0,0500 | (-01192 - ,0192 | 0,9048 | 0,9093 | 0,9500 | 0,0000 | 0,8095 | 0,9048 | 0,0000 | N/A |
| Approach: Treatment | 0,8590 | (,5922 - 1,1259) | 0,9524 | 0,6621 | 0,8889 | 0,9697 | 0,9048 | -0,5714 | 0,0476 | 0,8591 |
| Inform of violence against men as a serious threat | 0,7380 | (,3951 - 1,0799) | 0,9048 | 0,6372 | 0,9375 | 0,8000 | 0,8095 | 0,5238 | 0,0000 | 1,0000 |
| Inform of psychological consequences | 0,8000 | (,5417 - 1,0583) | 0,9048 | 0,5238 | 0,9231 | 0,8750 | 0,8095 | 0,2381 | -0,0952 | 0,8000 |
| Inform of social consequences | 0,8090 | (5574 - 1,0607) | 0,9048 | 0,5011 | 0,9000 | 0,9091 | 0,8095 | -0,0476 | 0,0000 | 1,0000 |
| Inform of physical consequences | 0,9010 | (,7137 - 1,0891) | 0,9524 | 0,5170 | 0,9412 | 0,9600 | 0,9048 | -0,1905 | 0,0476 | 0,9014 |
| Inform of economic consequences | 0 | 0 | 1 | 0,9524 | 0 | 0,9756 | 0,9048 | 0,9524 | 0,0476 | 0 |
| Inform of legal consequences | N/A | N/A | N/A | N/A | N/A | N/A | N/A | N/A | N/A | N/A |
| Inform about powerlessness to act | 0,8056 | (,5493 - 1,0618) | 0,9048 | 0,5102 | 0,8889 | 0,9167 | 0,8095 | -0,1429 | 0 | 1 |
| Inform of how the issue is usually private | 0,8290 | (,5075 - 1,1510) | 0,9524 | 0,7211 | 0,8571 | 0,9714 | 0,9048 | -0,6667 | -0,0476 | 0,8293 |
| Inform of LGBTI stressors | 0 | 0 | 0,9524 | 0,9524 | 0 | 0,9756 | 0,9048 | 0,9524 | 0,0476 | 0 |
| Inform of formal support | 0,774 | (,3534 - 1,1949) | 0,9524 | 0,7891 | 0,973 | 0,8 | 0,9048 | 0,7619 | -0,0476 | 0,7742 |
| Recommended Response | 0 | 0 | 0,9048 | 0,9048 | 0,95 | 0 | 0,8095 | -0,9048 | -0,0952 | N/A |
| Efficacy of the Recommended Response | ,8108 | (,5659-1.0557) | ,9048 | ,4966 | ,9091 | ,9 | ,8095 | -,0476 | -,00952 | ,8108 |
| Severity of Threat | 0,64 | (,2881 - ,9919) | 0,8571 | 0,6032 | 0,9032 | 0,7273 | 0,7143 | 0,4762 | 0,1429 | 0,64 |
| Explicit Framing of Violence | 0,811 | (,5659 - 1,0557) | 0,9048 | 0,4966 | 0,9 | 0,9091 | 0,8095 | -0,0476 | -0,0952 | 0,8108 |
| Implicit Framing of Violence | 0,715 | (,4176 - 1,0123) | 0,8571 | 0,4989 | 0,8571 | 0,8571 | 0,7143 | 0,0000 | 0,0476 | 0,905 |
| Self-Efficacy | 0,807 | (,5581 - 1,0566) | 0,9048 | 0,5057 | 0,9167 | 0,8889 | 0,8095 | 0,1429 | 0,0952 | 0,8073 |
| Susceptibility to Threat | 1 | 1 | 1 | 0,6916 | 1 | 1 | 1 | -0,6190 | 0 | 1 |
| Change behavior | 0,691 | (,2924 - 1,090) | 0,9048 | 0,6916 | 0,9412 | 0,75 | 0,8095 | 0,6190 | 0 | 1 |
| Change attitudes and beliefs | 0,767 | (,4608 - 1,0726) | 0,9048 | 0,5918 | 0,9333 | 0,8333 | 0,8095 | 0,4286 | 0 | 1 |
| Well-being despite social norms | 0,417 | (-,0011 - ,8344) | 0,8095 | 0,6735 | 0,5 | 0,8824 | 0,619 | -0,6190 | -0,1905 | 0,4167 |
| Need to change gender roles | 0,829 | (,5075 - 1,1510) | 0,9524 | 0,7211 | 0,8571 | 0,9714 | 0,9048 | -0,6667 | 0,0476 | 0,8293 |
| Diss: Powerlessness vs. Empowerment | 0,769 | (,4732 - 1,0652) | 0,9048 | 0,5873 | 0,9333 | 0,8333 | 0,8095 | 0,4286 | -0,0952 | 0,7692 |
| Diss: Love vs. Abuse | 1 | 1 | 1 | 0,6916 | 1 | 1 | 1 | -0,6190 | 0 | 1 |
| Diss: Violence exclusively directed at women vs violence is genderless | 0,488 | (-,0071 - ,9827) | 0,8571 | 0,7211 | 0,5714 | 0,9143 | 0,7143 | -0,6667 | -0,0476 | 0,8293 |
| Colloquialisms | 1 | 1 | 1 | 0,5011 | 1 | 1 | 1 | -0,0476 | 0 | 1 |
| Violence represented: type of relationship not defined | 0,809 | (,5574 - 1,0607) | 0,9048 | 0,5011 | 0,9091 | 0,9 | 0,8095 | 0,0476 | 0 | 1 |
| Violence represented: different sex relationships | 0,877 | (,6441 - 1,1103) | 0,9524 | 0,6122 | 0,9091 | 0,9677 | 0,9048 | -0,0476 | 0,0476 | 0,8772 |
| Violence represented: same sex relationships | 0,618 | (,1530 - 1,0834) | 0,9048 | 0,7506 | 0,6667 | 0,9444 | 0,8095 | -0,7143 | -0,0952 | 0,6182 |
| Person represented: only the victim | 0,788 | (,5146 - 1,0611) | 0,9048 | 0,551 | 0,9286 | 0,8571 | 0,8095 | 0,3333 | -0,0952 | 0,7879 |
| Person represented: victim and perpetrator | 1 | 1 | 1 | 0,7551 | 1 | 1 | 1 | -0,7143 | 0 | 1 |
| Style: Demonstration of Suffering | 0,798 | (,5322 - 1,0639) | 0,9048 | 0,5283 | 0,9231 | 0,875 | 0,8095 | 0,2381 | 0 | 0,7981 |
| Style: Intimate portrayal | 0,807 | (,5581 - 1,0566) | 0,9048 | 0,5057 | 0,8889 | 0,9167 | 0,8095 | -0,1429 | -0,0952 | 0,8073 |
| Style: Reenactment of violence | 0,462 | (-,1366 - 1,0597) | 0,9048 | 0,8231 | 0,5 | 0,9474 | 0,8095 | -0,8095 | -0,0952 | 0,4615 |
